# Establishment of a Maternal Newborn Health Registry in the Belgaum District of Karnataka, India

**DOI:** 10.1186/1742-4755-12-S2-S3

**Published:** 2015-06-08

**Authors:** Bhalachandra S Kodkany, Richard J Derman, Narayan V Honnungar, Naresh K Tyagi, Shivaprasad S Goudar, Shivanand C Mastiholi, Janet L Moore, Elizabeth M McClure, Nancy Sloan, Robert L Goldenberg

**Affiliations:** 1 KLE University’s Jawaharlal Nehru Medical College, Belgaum, India; 2 Christiana Care Health Services, Newark, DE, USA; 3 RTI International, Durham, NC, USA; 4 Department of Obstetrics/Gynecology, Columbia University, New York, NY, USA

**Keywords:** India, pregnancy registration

## Abstract

**Background:**

Pregnancy-related vital registration is important to inform policy to reduce maternal, fetal and newborn mortality, yet few systems for capturing accurate data are available in low-middle income countries where the majority of the mortality occurs. Furthermore, methods to effectively implement high-quality registration systems have not been described. The goal of creating the registry described in this paper was to inform public health policy makers about pregnancy outcomes in our district so that appropriate interventions to improve these outcomes could be undertaken and to position the district to be a leader in pregnancy-related public health research.

**Methods:**

We created a prospective maternal and newborn health registry in Belgaum, Karnataka State, India. To initiate this registry, we worked with the Ministry of Health to first establish estimated birth rates and define the catchment areas of the clusters, working within the existing health system and primary health centers. We also undertook household surveys to identify women likely to become pregnant. We then implemented monitoring measures to ensure high quality and completeness of the maternal newborn health registry. All pregnant women in the catchment area were identified, consented and enrolled during pregnancy, with follow-up visits to ascertain pregnancy outcomes and mother/infant status at 42-days postpartum.

**Results:**

From 2008 through 2014, we demonstrated continued improvements in both the coverage for enrollment and accuracy of reporting pregnancy outcomes within the defined catchment area in Belgaum, India. Nearly 100% of women enrolled had follow-up at birth and 99% had 42-day follow-up. Furthermore, we facilitated earlier enrollment of women during pregnancy while achieving more timely follow-up and decreased time of reporting from the date of the pregnancy event.

**Conclusions:**

We created a pregnancy-related registry which includes demographic data, risk factors, and outcomes allowing for high rates of ascertainment and follow-up while working within the existing health system. Understanding the elements of the system used to create the registry is important to improve the quality of the results. Tracking of pregnancies and their outcomes is an important step toward reducing maternal and perinatal mortality.

## Introduction

Globally, it is estimated that about 289,000 maternal deaths, 2.6 million stillbirths, and 2.9 million neonatal deaths currently take place each year [1-3]. The majority of these deaths occur in areas where vital registration systems do not exist or are poorly functioning. This lack of appropriate data may result in a large underestimation of the number of maternal, fetal and neonatal deaths that are reported. While many publications exist on the use of registries to capture vital events, sparse information is available on the development of reliable registration systems to register pregnancies, capture birth outcomes (including maternal and perinatal deaths), as well as to determine the cause of death. Such information is key to informing programs likely to have the greatest public health impact. Availability of reliable data may have implications for focused education and economic planning that extend beyond the health sector. In this paper we describe a registry created in Belgaum, Karnataka, India. The goal of creating the registry was to inform public health policy makers about pregnancy outcomes in our district so that appropriate interventions to improve these outcomes could be undertaken and to position the district to be a leader in pregnancy-related public health research.

Vital registration, with a long and rich history in the United States and Western Europe, has been critical to the documentation of incidence and causes of maternal, fetal and neonatal death in these regions. Additionally, vital registration systems have proven value in the identification of factors to which temporal changes in pregnancy-related deaths may be associated [4]. Such registration systems may also prove critical in accurately reflecting the rate of progress towards country health and health system goals, such as the United Nations Millennium Development Goals [5-8].

Where functional vital statistics registries do not exist, countries may estimate births and deaths through suboptimal mechanisms such as surveys. These surveys are done on a periodical basis and thus make tracking of trends difficult. Furthermore, they are often comprised of small numbers and thus have estimates of uncertainty with wide confidence intervals, particularly for maternal, fetal and neonatal mortality [9]. If near complete registration is not attained, under-reporting of important events is likely to occur. India is one of the most populous countries with among the highest numbers of maternal and newborn deaths. Misinformation on births and deaths in India will likely have a significant impact on global estimates.

## History of vital registration in India

In India, vital statistics data are available from four major sources: (1) Indirect Estimates from the Decennial Census first initiated in 1881 (2) The Civil Registration System (CRS) which was initiated in 1958; (3) The Sample Registration System (SRS) which began in 1970 and, finally, (4) The National Family Health Survey (NFHS) which was initiated in 1992 [10-14]. The first three are operated by the Registrar General under the Ministry of Home Affairs in India. The NFHS functions under the Ministry of Health and Family Welfare of the Government of India. (MOH) The Registration of Birth and Deaths Act became operational in 1967, twenty years after India’s independence, and was extended to the entire nation in 1969 resulting in the SRS. The SRS registers the events of birth and deaths in some but not all areas of India. In this system, vital events have been registered using a dual recording system. Initially, the resident enumerators record all births and deaths within their assigned geographic area. Thereafter, a full time supervisor collects information during a house-to-house confirmatory survey. The events collected by both methods are matched, and those unmatched are then re-verified to eliminate errors of missing or duplicate events. Statistics from these programs may continue to be appropriate for framing the national health policy and guidelines. However, they may not be sufficiently robust to generate policies that have the desired impact on improving public health programs, especially in rural areas wherein 72% of the Indian population resides [15].

In 1951, India added family planning into its national health care policy to address individual needs and also to contain its population growth. Consequently, the primary health center (PHC) was established as the service nodal point for identifying couples that required birth control services and was incorporated into the “Eligible Couple Survey Register” (ECSR). These registers are still maintained at the PHCs and sub-centers.

In Karnataka, India, the registration of births and deaths became operational throughout the state beginning in 2000. Healthcare providers serving the district health system and other officials at the village and sub-district levels collect vital events information. While, the accuracy and completeness of these government statistics are perceived to be relatively good, they are not as complete as many formal functioning pregnancy and vital statistics registries in more developed countries. Of note, abortions and stillbirths, especially, are likely to be under-reported and/or misclassified.

In 2000, the U.S. *Eunice Kennedy Shriver* National Institutes of Child Health and Human Development (NICHD) funded the Global Network for Women’s and Children’s Health Research, incorporating a site based in Belgaum in Karnataka State, India. Among the research initiatives funded was the First Breath trial which was implemented in Belgaum in collaboration with the District Health System [16]. For the trial, all birth attendants were trained to implement the World Health Organization’s (WHOs) Essential Newborn Care program, and were randomized to receive resuscitation training. In the trial catchment areas, pregnant women were enrolled at delivery and followed up at days 7 and 28 postpartum. The data collection system for this study formed the basis for the pregnancy registry described below, although we emphasize that nearly all the components needed to develop the registry system were in place prior to the NICHD study.

## Study objective

The Maternal Newborn Health Registry (MNHR) is a prospective, population-based observational study that was initiated to better understand and quantify trends in outcomes of pregnancy and childbirth in rural PHCs clusters so as to provide data on abortions, stillbirths, newborn and maternal mortality [17,18]. This article details the methodology adopted to establish the PHC-based MNHR for recording all pregnancies and their outcomes occurring within, and outside, the designated geographical areas starting in May, 2008 and continuing to the present. We also present results on the indicators of quality of the data over this time period.

## Methods

### Registration of pregnancy

The MNHR receives inputs from the register of Married Women of Reproductive Age (MWRA, formerly known as the ECSR) at the PHCs and sub-centers. The MWRA register contains information regarding couples eligible to receive contraception services, those who are infertile and those yet to reach menopause. It also identifies those couples wishing to, and who are likely to conceive, within the following year. The MWRA is updated every year by a house-to-house survey (occurring over a 3-months period) conducted by auxiliary nurse midwives (ANMs), Anganwadi workers (AWWs) and accredited social health activists (ASHAs). In addition to informing public health needs, the MWRA provides early identification for pregnancies and informs enrollment in the MNHR. Building on the MWRA, the data collection for the MNHR began in May 2008 in 20 geographic areas, each incorporating a PHC with ≥300 annual deliveries and covering a total population of about 660,000, which is also encompassed in the MWRA catchment area. Although one of the primary sources used to identify pregnant women is the MWRA, registry staff and other health workers attempt to identify all pregnant women, married or not, in the catchment area and these women are included in the registry.

### Inclusion criteria for the Global Network MNHR

In central and southern India, where Karnataka is located, it is common, particularly among nulliparous women, to stay with relatives and deliver where those relatives reside. Similarly, pregnant women may enter the catchment area to deliver at the home of relatives. Recording movement of pregnant women for delivery was one of the major problems we had to overcome to approach 100% ascertainment. Thus, pregnant women were eligible for inclusion in the MNHR if they were residents of the study clusters during pregnancy, regardless of delivery location. Women were also eligible if they lived outside the geographic area but delivered within the MNHR catchment area. All identified women who met criteria were included in the registry after they had provided written informed consent.

### Enrollment and follow-up

All women identified as likely to conceive were followed up by monthly visits through the MWRA. After a missed menstrual period and a pregnancy confirmed by a pregnancy test, an eligible woman is then invited to be enrolled in the MNHR by ASHA workers. Once the woman has been enrolled, the MNHR registry administrators (RAs), who are medical officers of the PHCs, obtain consent and complete data entry forms for the MNHR. This methodology of screening for pregnancy has also enabled us to document spontaneous abortions and medical terminations of pregnancy (MTPs) at the community level. These data had not been previously collected.

### Data forms

**Figure 1:**
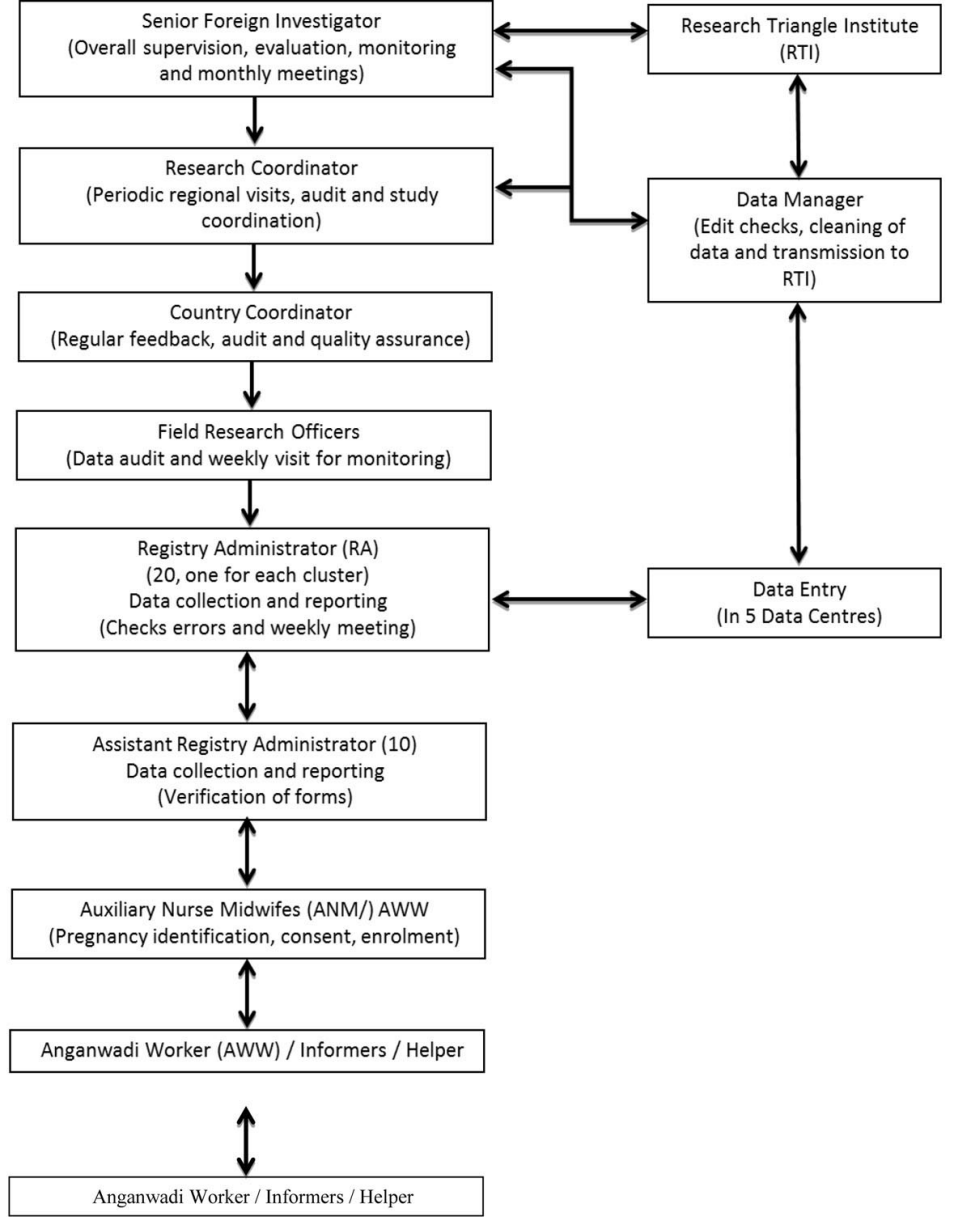
Administrative organization of the Maternal Newborn Health Registry

Three forms are used to collect study data, are available elsewhere [18]. (1) The first, filled out as soon as possible after conception and enrollment, collects the maternal demographic data and the date of last menstrual period; (2) A perinatal form, completed at the time of pregnancy loss or delivery, and information relative to the status of antenatal care, maternal health issues, the mode, time and place of delivery, characteristics of the fetus/neonate, and any adverse events at or soon after the delivery; (3) At the 6 week follow-up visit, a third form contains information about the status of the newborn and the mother through 42 days post-delivery, and includes information on any major adverse events occurring between birth and the follow up visit. Protocol deviations, withdrawals from the study, loss to follow-up and time lag between event, data collection, entry and transmission to the Data Management Center are also assessed and recorded. The overall administrative structure of the MNHR is shown in Figure 1.

### Data collection procedures and staffing

After the forms obtained at the three specific times discussed above are compiled by the RA, the completed forms are then verified and collected by the data entry operators on fixed days of the week. Data are entered into an electronic database at regional data centers located at the sub-district level and transmitted to a central data management center. The quality of data received is regularly monitored by a research coordinator, who oversees the study, and field research officers, who are generally medical officers with oversight of field activities, together with RAs and data entry operators at the regional data centers. Cleaning and editing data and transmission to the Global Network Data Coordinating Center (DCC), located at a central location in the U.S. are ongoing activities. Combined data are processed and analyzed at the DCC for data consistencies and completeness. The DCC sends monthly monitoring reports which are discussed by the study team (i.e., the data manager, RA’s and field supervisors) and serve as process and outcome indicators. Inconsistencies and unexpected findings or trends, when they occur, are verified and corrective steps initiated during monthly meetings conducted at the study site headquarters in Belgaum.

### Quality Assurance and Completion of Data Collection Instruments

Data quality was monitored in a number of ways. The monitoring metrics included: 1) the number of days taken to complete the forms and record of all relevant events, 2) the proportion of women consented for inclusion in the study from the total of pregnancy women screened who were eligible, 3) the rates of MTP, miscarriages, stillbirths and live births, as well as 4) the proportion of follow-up through 42 days postpartum. The monthly monitoring reports contain these elements which allow the team to further check and validate the results.

An important feature of the quality measurement is having an estimate of the number of births that should occur in each cluster. We use an estimate of the crude birth rate (CBR) within the study clusters as an indicator of completeness of registration of pregnancy outcomes and as a check to ensure complete capture of all births. The CBR was computed from the live births and the estimated population of the cluster. Significant differences of the number of births registered from those expected are investigated.

The time taken in completion after the event of interest and its entry into the data management system has also been considered as a quality indicator, along with documentation of the extent of birth weights recorded and the presence of a healthcare provider at the time of delivery.

## Results

Between 2008 and 2014, the MNHR catchment area comprised a total population of approximately 660,000 (Table 1). Of these, approximately 120,000, or 183 per 1000 population, were women of reproductive age. During that time, each year there were between 11,800 and 15,100 residents enrolled in the MNHR as well as between 4,400 and 7,900 enrolled women who lived outside the study area. Thus, about 31% of all women enrolled lived outside the study area but nevertheless delivered within the area.

**Table 1:** Study area population, residents and non-residents in the Maternal Newborn Health Registry and crude birth rates for residents, by year, 2008 - 2013

Year (May-April)	Population of study area	Residents in MNHR (N)	Crude birth rate (%)	Non-residents In MNHR, (N)	Non-residents In MNHR (%)
2008 –09	655,676	15,105	23.0	4,438	22.7

2009 –10	648,436	14,364	22.2	5,920	29.2

2010 - 11	649,899	14,730	22.7	7,330	33.2

2011 - 12	655,189	14,504	22.1	7,587	34.3

2012 - 13	662,317	14,470	21.9	7,931	35.4

Early registration of pregnancy (less than 12 weeks gestation) among all those enrolled in the MNHR increased from 5.9% in 2008-09 to 34.0% in 2013-14 (Table 2). In addition, registration at 12-20 weeks among all women also increased from 19.9% to 28.5% in 2013-14. Conversely, enrolment >20 weeks decreased from 74.2% to 37.5%.

**Table 2:** Gestational age at registration of all pregnancies in the study area Maternal Newborn Health Registry by year, 2008-2014

	Total	May 2008 -April 2009	May 2009 -April 2010	May 2010 -April 2011	May 2011 -April 2012	May 2012 -April 2013	May 2013 -April 2014
N	123,188	19,311	20,212	22,020	22,061	22,392	17,192

< 12 weeks, N (%)	24,702 (20.1)	1,144 (5.9)	3,482 (17.2)	4,382 (19.9)	4,642(21.0)	5,211 (23.3)	5,841 (34.0)

12 - 20 weeks, N (%)	36,647 (29.7)	3,845 (19.9)	6,938 (34.3)	7,392 (33.6)	6,709 (30.4)	6,866 (30.7)	4,897 (28.5)

> 20 weeks, N (%)	61,839 (50.2)	14,322 (74.2)	9,792 (48.4)	10,246 (46.5)	10,710 (48.5)	10,315 (46.1)	6,454 (37.5)

**Figure 2:**
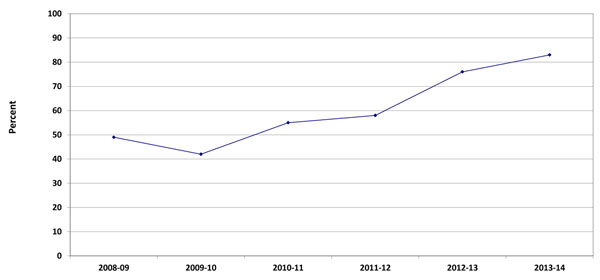
Percent of women in the Maternal Newborn Health Registry with a postpartum visit within one week of delivery by year, 2008-2014

**Figure 3:**
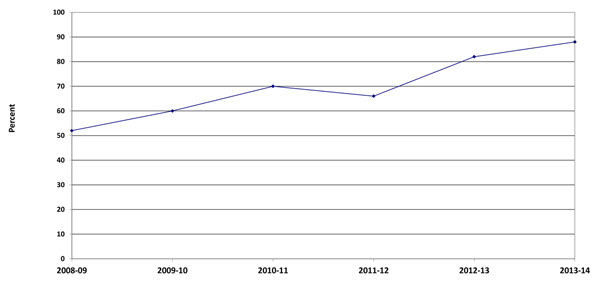
Percent of women in the Maternal Newborn Health Registry with a 42-day follow-up visit within 2 weeks of expected date by year, 2008-2014

Timely reporting is one of the hallmarks of a quality data collection system. Ongoing process evaluation and quality improvement measures are reflective of documented changes over time. In 2008-09, 48.8% of the birth forms were completed within one week of delivery. This rate improved to 83.3% in 2014 (Figure 2). Timeliness of the completing the 42-day visit is addressed in Figure 3. Visits which were conducted within one week of 42-days increased from 52.0% in 2008-09 to 89.7% in 2013-14.

**Figure 4:**
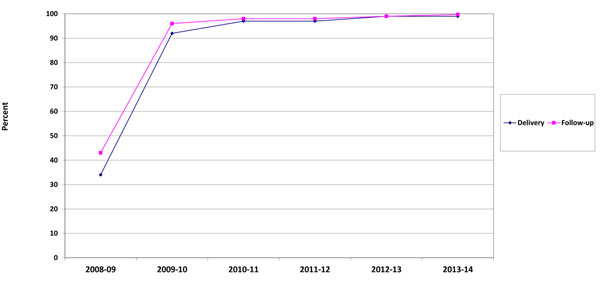
Percent of women in the Maternal Newborn Health Registry with complete data entry within one week of study visit for delivery and 42-day follow-up, by year, 2008-2014

The benchmark for entering data within a 2 week window after the birth visit was not considered satisfactory during 2008-09 as only 34.4% of the delivery data forms were keyed in that time period (Figure 4). However, in subsequent years, data entry within 2 weeks improved to 99.4%. The same pattern was observed for the data entry for the 42-day visit, with entry increasing from 42.9% to 99.7%.

Birth weight recording also improved significantly from a rate of missing birth weights of 10.1% in 2008-09 to only 0.8% in 2013-14. Missing birth weights collected on neonatal deaths totalled 11.1% in 2008-09 which were reduced to 2.0% in 2013-14. Similarly, missing birth weight for stillbirths, reported at 48.2% in 2008-09, was reduced to 32.1% in 2013-14 (Table 3).

**Table 3:** Rates of missing birth weight in the Maternal Newborn Health Registry for total births, neonatal deaths and stillbirths by year, 2008-2014

	2008 -09	2009 -10	2010 -11	2011 -12	2012 -13	2013 -14
Deliveries N	19,013	19,395	20,901	21,112	21,185	16,151

Measured Birth Weight Missing N (%)	1,911(10.1)	585(3.0)	295(1.4)	176(0.8)	165(0.8)	132 (0.8)

Neonatal Deaths N	459	524	559	473	524	393

Measured Birth Weight Missing N (%)	51(11.1)	40(7.6)	18(3.2)	7(1.5)	11(2.1)	8 (2.0)

Stillbirths N	641	589	564	491	536	336

Measured Birth Weight Missing N (%)	309(48.2)	219(37.2)	148(26.2)	115(23.4)	119(22.2)	108 (32.1)

Finally, over the 5 years of the MNHR, Table 4 shows that virtually all those women who were screened, then consented to be included in the MNHR, and of those pregnant women consented, virtually all had pregnancy outcomes and 42 day follow-up data collected.

**Table 4:** Follow-up rates among pregnancies screened in the Maternal Newborn Health Registry in Belgaum, India, 2008 - 2014

Events	Number	%
Screened, N	123,711	-

Consented, N (% of screened)	123,659	99.96

Pregnancy outcome obtained (% of consented)	123,581	99.94

Follow-up until 42 days of delivery (% of consented)	123,354	99.81

In summary, from the very inception, the MNHR has been embedded within the health care structure of the MOH and has sought to utilize and strengthen the available systems and tools. The household survey, listing of married women of reproductive age, early pregnancy registration and tracking the outcomes of pregnancies are mandated activities of the MOH as per the national Reproductive and Child Health program. We have attempted to simplify these activities and have provided performance based incentives to the health workers, measures that have been subsequently adopted by the MOH as well. Periodic training and regular supportive supervision have enabled effective implementation of the registry. A strong collaboration has been forged between the academic faculty and the MOH officials at every level for mutual benefit.

## Discussion

The methodology adopted for the MNHR allowed tracking of 99.95% of the pregnancies identified in our study area along with collection of pregnancy outcomes. Earlier registration permitted capturing more robust data on spontaneous abortions, MTP, stillbirths and maternal and neonatal deaths. The MNHR also captured the pregnancy outcomes for mothers who, often for cultural reasons, relocated to deliver elsewhere. We were also able to capture data on women who came from afar to the catchment area for their delivery. This registry may differ from other registries which are limited to capturing events occurring only in a specific geographical area, and therefore may not be representative of the population living in that area.

Modifying the existing MWRA to include current pregnancies and those women likely to conceive in the following year was an important innovation that facilitated the capture of 99.9% of consented pregnancies and their outcomes through 6 weeks postpartum. This relatively high predictive value has been an enabler to allow for pre-conception and early first trimester inclusion in clinical trials.

In order to ensure quality of the data collected, process measures were put into practice and analyzed. These included: (1) cluster-wide consistency checks; (2) monitoring time lag in completing the data collection from occurrence of the event to its subsequent electronic data entry, as well as; (3) identifying missing birth weights, reflected in accurate recording of birth weight in more than 94% of all births and more than 99% of neonatal deaths. To assure the completeness of the data, a comparison of expected number of pregnancies with actual number of pregnancies was monitored.

A question that might arise in the reader’s mind is whether a system such as that described in this paper is replicable in the rest of India. In India, the types of government workers mobilized for registry data collection in Belgaum are also available in each district throughout the country. Similarly, the systems upon which the MNHR was built are generally present. Therefore, we believe that if there is political will to carefully collect quality data, and a motivating entity such as the university staff and the MOH personnel in the Belgaum area, a similar registry could be created elsewhere. As a start, the MOH has implemented an electronic Mother Child Tracking System, modelled on the MNHR, nationally to monitor the outcomes of pregnancies.

There are a number of lessons learned from the creation of the MNHR in this area of India. Most important, is that from the beginning, this registry was a joint effort by the research group at the university hospital and the public health infrastructure of the MOH. Research-quality data was perceived as crucially important to both groups. Second, because both groups wanted information on both the births in the resident population as well as the births occurring in the geographic area regardless of residency of the mother, strong efforts were made to capture both types of births. This information is especially useful to health planners and providers in and out of the study area. Third, it became obvious that triangulating outcomes in the various ongoing government registries as a check on data in the MNHR was crucial in getting as close to complete registration as possible. Fourth, the use of the CBR as a way to estimate the expected number of births in each cluster was a very useful check to help insure completeness of registration. Fifth, the use of the existing MWRA registry is an important feature of the system. By knowing who is likely to conceive in the near future combined with frequent surveillance, the MNHR administrators can determine pregnancy outcomes, including miscarriages and MTPs, which historically have been poorly collected in many geographic areas.

There have been difficulties in managing the MNHR as well. Finding and retaining trained staff at every level and sustaining their motivation year after year has been a continuing challenge. Shortages of physicians and other trained personnel has been a problem. In part because of an increased burden posed on them by a multitude of national programs, the PHC staff have had less time to perform their registry duties and thus over the years, independent staff have been increasingly recruited to function as RAs. With staff turnover, training and retraining of staff has been an important feature of the MNHR and has helped to ensure data quality and timeliness. Most important in the creation and maintenance of the MNHR was the practice of holding frequent meetings with stake-holders including the rural community, the district health officials and the MOH of Karnataka State to address concerns as they arose.

Use of the MWRA registry also is a crucial tool for identifying women for enrollment into pre-pregnancy studies as well as studies requiring first trimester enrollment. Since the norm in many low resource settings is for women to enroll in prenatal care at 20 weeks or later, use of the MWRA registry enables studies to be undertaken that otherwise would be difficult or impossible in these settings. Finally, this paper describes some of the measures used to monitor the quality of the data collected over the time the MNHR has been in existence. These include measures such as the percent of pregnancies registered in the first trimester, the timeliness of reporting within specified limits, and the amount of missing data such as measured birth weights for live births and stillbirths. Improvement in many of these metrics was documented. The ongoing comprehensive monitoring of a number of quality metrics allows the NMHR administrators to have good faith that the data received from this site is of exceptional quality.

In summary, this report details many of the steps that need to be taken in any low-resource area without a good vital statistics system to establish a credible pregnancy outcome registry. We believe these steps are applicable anywhere there is a desire to really understand which residents in a geographic area are getting pregnant as well as who is delivering in the area, and the outcome of their pregnancies. An ongoing monitoring system better allows the registry administrators and interested reviewers to understand the quality of the data. Knowledge regarding the reproductive health status in the registry should identify key areas for implementation of evidence-based interventions that will assist in meeting India’s Newborn Action Plan and help achieve single digit neonatal mortality and stillbirth rates by 2030 [5]. While cause and effect is difficult to prove, improvement in a number of pregnancy outcomes have occurred in Belgaum since the MNHR was established and these are documented in other papers in this supplement [19-21].

## Conflict of interest

The authors declare that they have no conflict of interest.

## Statement of authorship

BSK conceived the paper and wrote the first draft; EMM, RLG, RJD and SSG participated in writing and RLG, SSG, EMM and NLS edited the draft. NVH, NKT, SSG, and SCM monitor study data collection and analyses. JLM conducted study analyses with EMM. All authors reviewed and approved the final manuscript.

## Peer review

Reviewer reports for this article can be found in Additional file 1.

## Supplementary Material

Additional file 1 Click here for file

## Declarations

This article has been published as part of *Reproductive Health* Volume 12 Supplement 2, 2015: Research reports from the NICHD Global Network for Women’s and Children’s Health Research Maternal and Newborn Health Registry. The full contents of the supplement are available online at http://www.reproductive-health-journal.com/supplements/12/S2. Publication of this supplement was supported by grants from the Eunice Kennedy Shriver National Institute of Child Health and Human Development to RTI International.
