# Supplementary material for: Establishment of a Maternal Newborn Health Registry in the Belgaum District of Karnataka, India
Source: Reprod Health. 2015 Jun 8;12(Suppl 2):S3. doi: 10.1186/1742-4755-12-S2-S3 (PMC4464217; doi:10.1186/1742-4755-12-S2-S3)
Supplement: Additional file 1 [file 1742-4755-12-S2-S3-S1.pdf]

Referee's comments to the authors– this sheet WILL be seen by the author(s) and published with the article

Title	Estalishment of a Maternal Newborn Health Registry in the Belgaum District of Karnataka, India
Author(s)	Bhalachandra S Kodkany, Richard J Derman, Narayan Honnungar, Naresh Tyagi, Shivaprasad S Goudar, Mastiholi S, Janet L Moore, Elizabeth M McClure, Nancy Sloan, Robert L Goldenberg
Referee's name	Neal Brandes

When assessing the work, please consider the following points, where applicable:

1. Is the question posed by the authors new and well defined?
2. Are the methods appropriate and well described, and are sufficient details provided to replicate the work?
3. Are the data sound and well controlled?
4. Does the manuscript adhere to the relevant standards for reporting and data deposition?
5. Are the discussion and conclusions well balanced and adequately supported by the data?
6. Do the title and abstract accurately convey what has been found?
7. Is the writing acceptable?

Please make your report as constructive and detailed as possible in your comments so that authors have the opportunity to overcome any serious deficiencies that you find and please also divide your comments into the following categories:

- Major Compulsory Revisions (which the author must respond to before a decision on publication can be reached)
- Minor Essential Revisions (such as missing labels on figures, or the wrong use of a term, which the author can be trusted to correct)
- Discretionary Revisions (which are recommendations for improvement but which the author can choose to ignore)

Where possible please supply references to substantiate your comments.

When referring to the manuscript please provide specific page and paragraph citations where appropriate.

General comments:
This article is documentation of the work of an experienced global research team that has established strong surveillance sites and procedures as part of the Global Network. The results are quite impressive, but also not entirely surprising. As a reader interested in both research methods and implementation issues I would like to know a bit more how they achieved the results. Specifically, how do they see their approach vis-à-vis other established surveillance sites, e.g. INDEPTH network,. What were the challenges they had to overcome to achieve coverage over the last few years.?

From the implementation/programmatic side I would like to know more how they collaborated with the ministry of health, the challenges in academia-MOH joint activities, and whether any of their tools or procedures were adopted by the local health authorities in areas ajoining the surveillance areas. In the area the research team worked how integrated or separate is their data system and workers vis-à-vis the MOH? What are lessons learned. A sidebar box or table of challenges and their approaches to solutions would be most appreciated.

Finally, providing a web annex with some of the tools they used might be useful learning tools for other researchers and program implementers

If the authors can address the issues above readily I would encourage them to revise the paper. Otherwise I am supportive of the publication in the current form.

Major compulsory revisions:

Please clarify on p. 21 Figure 2 whether the reference is to delivery or postpartum visits.

Minor essential revisions:

Referee's comments to the authors– this sheet WILL be seen by the author(s) and published with the article

Title	Estalishment of a Maternal Newborn Health Registry in the Belgaum District of Karnataka, India
Author(s)	Bhalachandra S Kodkany, Richard J Derman, Narayan Honnungar, Naresh Tyagi, Shivaprasad S Goudar, Mastiholi S, Janet L Moore, Elizabeth M McClure, Nancy Sloan, Robert L Goldenberg
Referee's name	Stan Edlavitch

When assessing the work, please consider the following points, where applicable:

1. Is the question posed by the authors new and well defined?
2. Are the methods appropriate and well described, and are sufficient details provided to replicate the work?
3. Are the data sound and well controlled?
4. Does the manuscript adhere to the relevant standards for reporting and data deposition?
5. Are the discussion and conclusions well balanced and adequately supported by the data?
6. Do the title and abstract accurately convey what has been found?
7. Is the writing acceptable?

Please make your report as constructive and detailed as possible in your comments so that authors have the opportunity to overcome any serious deficiencies that you find and please also divide your comments into the following categories:

- Major Compulsory Revisions (which the author must respond to before a decision on publication can be reached)
- Minor Essential Revisions (such as missing labels on figures, or the wrong use of a term, which the author can be trusted to correct)
- Discretionary Revisions (which are recommendations for improvement but which the author can choose to ignore)

Where possible please supply references to substantiate your comments.

When referring to the manuscript please provide specific page and paragraph citations where appropriate.

General comments:

The investigators convincingly present their efforts to create a quality comprehensive Maternal Newborn Health Registry in the Belgaum District of Karnataka State, India. Their methodology is well documented and the article is well written.

However, the authors go beyond reporting their experience in establishing the registry and suggest that their project provides a model for low resource areas with good vital statistics systems to accomplish tracking of pregnancies and health outcomes. The premise being that establishing a registry is as an important step toward reducing maternal and perinatal mortality.

These efforts were substantially (not indicated if fully) by the US NICHD. The conceptualization and research team is international and includes a US data center. As they note, it is based on the collaboration of KLE University's Jawaharlal Nehru Medical College, Belgaum, India, and the Ministry of Health Karnataka.

1. Can similar registries be established in other areas of India or other low-resource countries without this infusion of external funding and strong academic center involvement?
2. The local system in the Belgaum District includes several levels of trained personnel. Does the public health system in other states in India include the dame density of auxiliary nurse midwives (ANMs), Anganwadi workers

(continue on the next sheet)

1. *Continued*: The primary reason for establishing a newborn health registry is to improve public health and lower maternal and infant mortality rates and decrease morbidity and associated baby and mother complications. Since this effort has been successfully implemented in the Belgaum District for at least 2008, what measures of health success have been evaluated? How have they been evaluated and what were the findings.

Major compulsory revisions:

The authors need to include information on the impact of this registry on maternal and infant health in the Belgaum District. Is this a labor intensive effort which cannot be replicated in other developing country situations?

These efforts were substantially (not indicated if fully) by the US NICHD. The conceptualization and research team is international and includes a US data center and has received scientific advice from global experts who are part of Global Network for Women's and Children's Health Research. As they note, it is based on the collaboration of KLE University's Jawaharlal Nehru Medical College, Belgaum, India, and the Ministry of Health Karnataka.

1. Can similar registries be established in other areas of India or other low-resource countries without this infusion of external funding and strong academic center involvement?
    a. What criteria needs to be met?
    b. Provide examples of Indian States, Districts and or other countries where this may be feasible without major external funding?
2. The local system in the Belgaum District includes several levels of trained personnel. Does the public health system in other states in India include the dame density of auxiliary nurse midwives (ANMs), Anganwadi workers (AWWs) and accredited social health activists (ASHAs) and Primary Health Centers (PHCs)?
3. The primary reason for establishing a newborn health registry is to improve public health and lower maternal and infant mortality rates and decrease morbidity and associated baby and mother complications. Since this effort has been successfully implemented in the Belgaum District for at least 2008, what measures of health success have been evaluated? How have they been evaluated and what were the findings.
4. The article does not address the issue of births to unmarried women. How many pregnancies can be identified in unmarried women or is this a non-issue in village India?

Optional:

1. What did this effort cost?
2. What are the recurrent costs of operating such a registry?

Supplement Editor's comments

Please state in the abstract the objective of your study.
Please references need some improvement: Reference 1, the name of the journal is lacking, Reference 7, please correct it. In references not coming from a Journal please provide the link and date accessed.
Please provide a better legend for figures. Each figure and it legend should be self-explicative. Do not use acronyms in legend for figures.
